# Blood pressures, heart rate and locomotor activity during salt loading and angiotensin II infusion in protease-activated receptor 2 (PAR_2_) knockout mice

**DOI:** 10.1186/1472-6793-8-20

**Published:** 2008-10-21

**Authors:** John J McGuire, Bruce N Van Vliet, Sarah J Halfyard

**Affiliations:** 1 Cardiovascular Research Group, Division of BioMedical Sciences, Faculty of Medicine, Memorial University, St. John's, Newfoundland, Canada

## Abstract

**Background:**

In this study we used radiotelemetry to measure hemodynamic variables and locomotor activity in conscious unrestrained male Protease-Activated Receptor 2 (PAR-2) knockout mice in order to provide a detailed assessment of their blood pressure phenotype. In addition we tested for an influence of PAR-2 on salt-sensitivity (8% versus 0.5% NaCl diet, 2.5 weeks) and angiotensin II-induced hypertension (1 μg Ile^5^-angiotensin II/kg/min versus 0.25 μl/h saline, 2 weeks).

**Results:**

Systolic arterial pressures of PAR-2 -/- (129 ± 1 mmHg, n = 21, P < 0.05) were statistically higher than those of C57BL/6J (124 ± 1 mmHg, n = 33) throughout the 24 h period under baseline conditions. Pulse pressures in PAR-2 -/- were also significantly elevated (33 ± 1 mmHg versus 30 ± 1 mmHg, P < 0.05), whereas diastolic arterial pressures were not. Heart rates in PAR-2 -/- were not significantly different than controls, with the exception that heart rate of PAR-2 -/- was 23 beats per min higher than controls (*P *< 0.001) during periods of nocturnal activity. The diurnal pattern and intensity of locomotor activity were not found to differ between strains. A high salt diet led to increased blood pressures, decreased heart rates, increased time spent active and decreased intensity levels of locomotor activity. Salt-induced changes in systolic and pulse pressures in PAR-2 -/- were less than in C57B/6J. Angiotensin II treatment increased pressures, decreased heart rates, decreased time spent active and decreased intensity levels of activity of PAR-2 -/-, all to the same extent as C57BL/6J. A trend of lower blood pressures during the middle period of angiotensin II treatment period was observed in individual PAR-2 -/-.

**Conclusion:**

The data indicated gene knockout of PAR-2 was associated with a modest change in blood pressure phenotype. PAR-2 -/- mice exhibited moderate elevation of systolic arterial and pulse pressures, yet no increased diastolic arterial pressure, no increased blood pressure responses to high salt diet and a subtle difference in the time course of the blood pressure responses to angiotensin II infusion.

## Background

Protease-activated receptor 2 (PAR_2_), a 7 transmembrane domain G protein coupled receptor, has been found to be activated endogenously by specific serine proteases [1,2]. Supposedly, rather than by the binding of any known soluble ligands in the circulation, serine proteases released by endothelial cells and other cells during inflammation and/or injury initiates PAR_2_-dependent actions that range from endothelial-dependent vasodilation to production of cytokines and stimulated *de novo *expression of adhesion molecules [3]. No obvious blood pressure phenotypes have been reported in PAR_2 _-/- albeit such *in vivo *observations have so far been limited to anaesthetized mice [4]. Studies have reported PAR_2 _-/- mice were resistant to induction of inflammatory disease models including arthritis [5,6], colitis [7], airway inflammation [8], and vascular acute inflammation [9,10] and renal inflammation [11]. On the other hand, PAR_2 _-/- were more susceptible to neurodegenerative inflammation [12,13] and focal brain ischemia [14]. Still in other studies, PAR_2 _-/- were no less or more affected than normal mice subjected to endotoxemia [15] and renal ischemia reperfusion injury [16]. To date there have been no studies *in vivo *using PAR_2 _-/- whereby their constitutive hemodynamics have been challenged such as in mouse models of acquired hypertension. High salt diet feeding [17] and angiotensin II infusions [18,19] can reveal novel pathophysiological interactions of receptor signaling on hemodynamic control of blood pressure. Serine proteinase activities (renal kallikreins in particular) have been linked to salt-sensitivity [20-22] and both salt- and angiotensin II-induced hypertension have been linked separately to pro-inflammation signalling pathways such as nuclear factor-κB shared in common with PAR_2 _[23-30]. Such findings provided a good rationale for testing the pathophysiological interactions of PAR_2 _with regulation of blood pressure during altered hemodynamic states particularly with the development of acquired hypertension.

The goals of this study were to describe the blood pressure phenotype of conscious PAR_2 _-/- using radiotelemetry. Specifically, we aimed to determine the baseline blood pressure level of PAR_2 _-/-, and whether PAR_2 _-/- were more salt-sensitive and/or more responsive to infusion with angiotensin II than controls. These data were expected to inform us about the interaction of PAR_2 _with blood pressure regulation and provide insight into therapeutic possibilities for PAR_2_-targetted drugs in the future.

## Methods

### Ethics

All protocols were approved by the Institutional Animal Care Committee of Memorial University in accordance with the guidelines and principles recommended for the use of animals in research by the Canadian Animal Care Council.

### Animals

Control mice (PAR_2 _+/+; C57BL/6J) and PAR_2_-deficient mice (PAR_2 _-/-; B6.Cg-*F2rl1*^tm1mslb^/J) were purchased from the Jackson Laboratories (Bar Harbor, ME). Except during high-salt (HS) diet feeding, mice were fed a standard regular-salt (RS) feed containing 0.28% sodium (0.5% NaCl), 1% calcium, 0.59% potassium and 0.93% phosphorus (NIH-31 autoclavable open formula mouse diet; Zeigler Bros Inc., PA). High-salt diet containing 8% NaCl was comprised otherwise as above (Zeigler Bros Inc., PA). Mice were provided water *ad libitum*. Mice were housed individually in air filter-topped cages in a room of the Animal Care Facility of the Health Sciences Center dedicated to recording radiotelemetry data. The room lighting was set to a 12 h light and 12 h dark cycle and room temperature was 20–22°C.

### Experimental Protocol

Mice (males; PAR_2 _+/+: 10–20 weeks of age, 25–30 g; PAR_2 _-/-: 10–30 weeks of age, 24–30 g) were implanted with a telemeter (TA11PA-C10, Datasciences Inc.) via the right carotid artery [31,32] under isoflurane/oxygen anaesthesia. All mice were allowed to recover from these surgeries for 13 to 18 days prior to recording their baseline variables. For the HS challenge protocol, the BP data were recorded by telemetry continuously for 3–9 days at baseline and then for 18 days following the switch to experimental diet (5 PAR_2 _+/+ and 5 PAR_2 _-/-). For the protocol comparing the effect of high Ang II in each strain, baseline recordings were made for a 4–7 day period. On Day 0 of the Ang II infusion protocol, mice were removed from their rooms shortly after the start of the light period and a micro-osmotic pump (ALZET model 1002) containing either isotonic saline (13 PAR_2 _+/+ and 7 PAR_2 _-/-) or Ang II (15 PAR_2 _+/+ and 9 PAR_2 _-/-; 1 μg Ile^5^-angiotensin/kg/min) was implanted dorsally s.c. Pump implantation (~10–15 min) was performed using isoflurane/oxygen anaesthesia, followed by monitoring their recovery for 4–6 h before being returned to their home cages. BP data sampling was then restarted ~18 h later at the start of the next light period (day 1). According to the manufacturer's literature provided with the product, steady state rate of infusion was expected to be reached 12 h after implant of micro-osmotic pumps. BP data in saline-treated and Ang II-treated mice were recorded for 12–13 days before being sacrificed. The proper placement of the catheter tips of telemeters in the aortic arch were confirmed at autopsy.

### Telemetry

Hemodynamic (systolic arterial pressure (SAP), diastolic arterial pressure (DAP), heart rate (HR)) and locomotor activity data were sampled for 3 s at 30 s intervals [32]. The 24 h data sets were processed using an Excel spreadsheet application designed for this purpose [33]. Data representing the inactive state for each mouse was defined by locomotor activity signal (Act, in arbitrary units) equal to 0 and active state was defined by Act > 0. Intensity of the continuous activity (Act intensity) was calculated by logarithmic base 10 transformation of all Act values when Act >0. Corrections for calibration drift were applied to data using the external pressure calibrations made at the end of the protocol [17,32]. Unexpected electrical power failures led to data exclusion and uneven sample sizes on several days of the experiment. Data recorded during cage change days (every 7 days) and day 0 of pump implants have been excluded. Only data sets from mice characterized by a stable 24 h MAP during the baseline recording period and pulse pressure (PP) ≥ 20 mmHg throughout the day were included in analyses. This criterion resulted in exclusion of telemeter data from 1 PAR_2 _+/+ and 4 PAR_2 _-/-. Regardless of the PP exclusion criterion, the recordings from these mice typically contained data points at both extremely high and low implausible ranges as well as periods of missing data acquisition signals, which have become characteristics used to identify artifacts in telemetry data.

### Data Analyses

Values reported and symbols on graphs are the means ± standard error of the mean. For overall characterization of phenotype, the data obtained at baseline preceding both experimental protocols (high salt feeding and micro-osmotic pump implants) were combined for each strain. Linear regression analysis indicated that age did not have an effect on baseline blood pressures or the effects of the experimental protocols in our study. The calculations of difference in telemetry variables during the experimental treatments are paired to the average value of the individual mouse's baseline recordings. There are 6 parts to the presentation of statistical analyses: 1) Comparison of the effect of strain on 12 h light and 12 h dark telemetry variables at baseline (2 way ANOVA). 2) Comparison of the effect of strain on 12 h light and 12 h dark variables at baseline with matching of variables to activity level (Act = 0 or Act >0; 2 way repeated measures ANOVA). 3) Comparison of the effect of strain on change in variables during HS diet (2 way repeated measures ANOVA). 4) Comparison of the effect of treatment (high salt vs normal salt) on variables with time (2 way repeated measures ANOVA). 5) Comparison of the effect of strain on change in variables during micro-osmotic pump treatments (2 way ANOVA). 6) Comparison of the effect of micro-osmotic pump treatment on variables (2 way ANOVA). Bonferroni post-hoc testing was used to identify differences between specific groups in the multiple comparisons. *P *< 0.05 was considered significant.

## Results

### Baseline hemodynamics of PAR_2 _-/- and PAR_2 _+/+ mice

**Figure 1:**
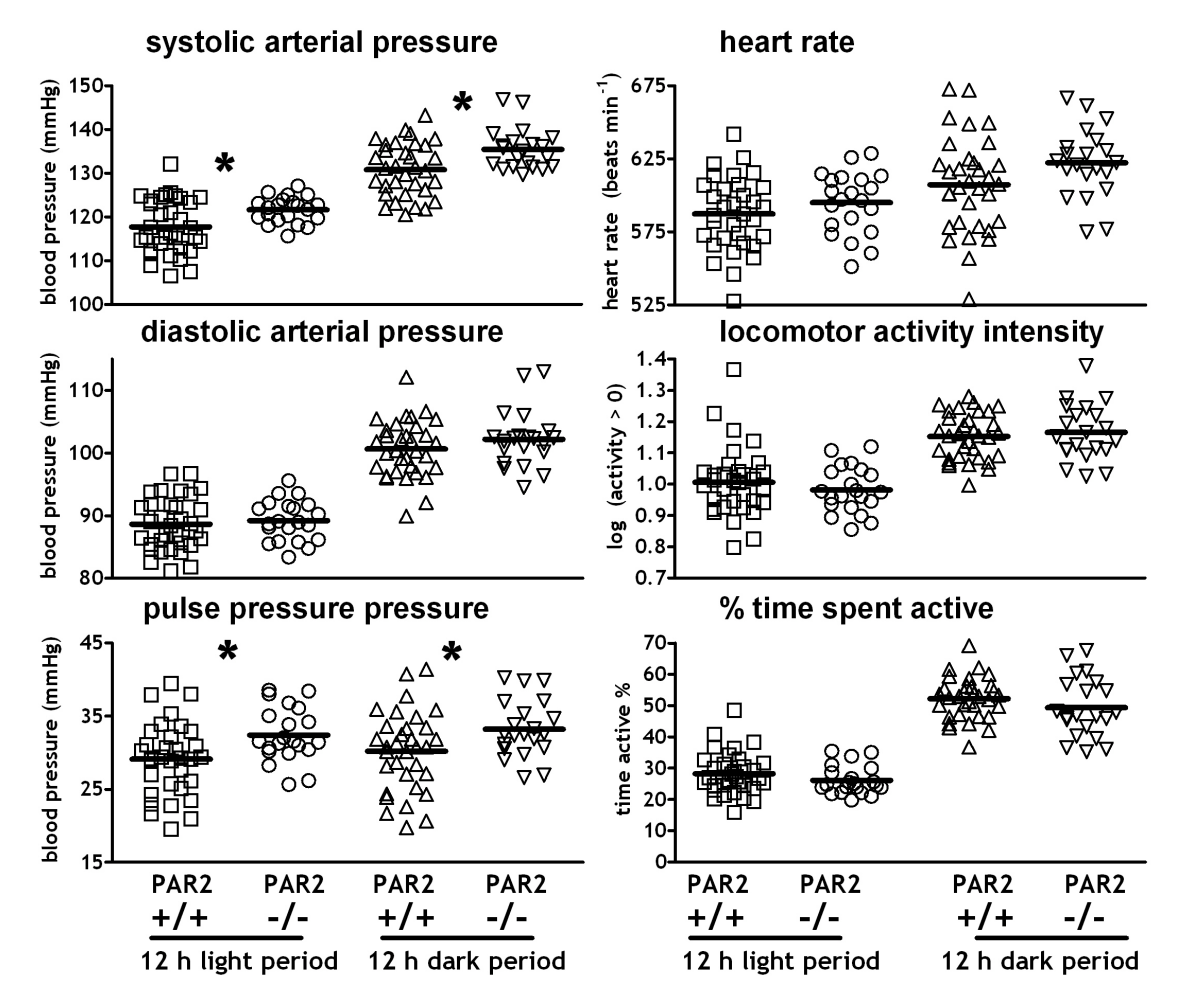
**Hemodynamics and locomotor activity in PAR_2 _-/- and PAR_2 _+/+**. Symbols are the means of variables from the 12 h light and 12 h dark periods in individual mice recorded via radiotelemetry during baseline periods of 2 days. The mean of each strain is indicated by a horizontal bar. n = 21 PAR_2 _-/- and 33 PAR_2 _+/+. * P < 0.05, PAR_2 _+/+ compared to PAR_2 _-/- using 2 way repeated measures ANOVA and Bonferroni post-hoc.

Blood pressures were modestly, but significantly (*P *< 0.05) higher in PAR_2 _-/- than PAR_2 _+/+ mice (Figure 1, Table 1). During the baseline period, SAP and pulse pressures (PP) of PAR_2 _-/- were ~3–4% and ~6–10% higher, respectively, than those of PAR_2 _+/+ for both 12 h dark and 12 h light periods. These differences between strains were independent of locomotor activity, being persistent during periods of both activity and inactivity (Table 2). In both strains, periods of locomotor activity were associated with higher blood pressures (12 h light: Δ SAP = 19 mmHg, Δ DAP = 17 mmHg; 12 h dark: Δ SAP = 13 mmHg, Δ DAP = 10 mmHg) (Table 2; *P *< 0.001). Differences in diastolic pressures and HR between PAR_2 _-/- and PAR_2 _+/+ were generally slight and, with one exception (active HR in dark period, P < 0.01) multiple comparisons between strains were not statistically significant (Table 2). Patterns of locomotor activity and the intensity level of activity during the light and dark periods also did not differ between strains (Figure 1, Table 2).

**Table 1:** Summary of baseline hemodynamics of PAR_2 _-/- and PAR_2 _+/+ mice.

**Time Period**	**Variable**	**PAR_2 _+/+**		**PAR_2 _-/-**	**Ratio PAR_2 _-/- to PAR_2 _+/+**
***12 h dark***	SAP (mmHg)	131 ± 1	**	136 ± 1	1.04
	DAP (mmHg)	101 ± 1		102 ± 1	1.00
	PP (mmHg)	31 ± 1	*	33 ± 1	1.06
	HR (beats min^-1^)	607 ± 6		622 ± 5	1.02

***12 h light***	SAP (mmHg)	118 ± 1	*	122 ± 1	1.03
	DAP (mmHg)	89 ± 1		89 ± 1	1.00
	PP (mmHg)	29 ± 1	*	32 ± 1	1.10
	HR (beats min^-1^)	587 ± 4		595 ± 5	1.01

Telemetry data from 12 hour light and 12 hour dark periods were obtained from 33 PAR_2 _+/+ and 21 PAR_2 _-/- mice. Values are mean ± standard error of mean. *P *values determined by multiple comparisons testing between strains during light and dark periods using 2 way ANOVA and Bonferroni post-hoc. *, *P *< 0.05; **, *P *< 0.01.

**Table 2:** Activity effects on hemodynamics of PAR_2 _-/- and PAR_2 _+/+ mice.

**Time Period**	**Variable**	**PAR_2 _+/+**		**PAR_2 _-/-**	**Ratio PAR_2 _-/- to PAR_2 _+/+**
***12 h dark***	% time active	52 ± 1		49 ± 2	0.96
	Act intensity	1.15 ± 0.01		1.17 ± 0.02	1.02
	SAP inactive (mmHg)	125 ± 1	*	128 ± 1	1.02
	SAP active (mmHg)	137 ± 1	***	142 ± 1	1.04
	DAP inactive (mmHg)	95 ± 1		96 ± 1	1.01
	DAP active (mmHg)	106 ± 1		109 ± 1	1.03
	PP inactive (mmHg)	29 ± 1	*	33 ± 1	1.14
	PP active (mmHg)	31 ± 1	*	34 ± 1	1.10
	HR inactive (beats min^-1^)	572 ± 6		581 ± 5	1.02
	HR active (beats min^-1^)	640 ± 6	**	663 ± 6	1.04

***12 h light***	% time active	28 ± 1		26 ± 1	0.93
	Act intensity	1.01 ± 0.02		0.98 ± 0.02	0.97
	SAP inactive (mmHg)	112 ± 1	*	117 ± 1	1.04
	SAP active (mmHg)	131 ± 1	**	136 ± 1	1.04
	DAP inactive (mmHg)	84 ± 1		85 ± 1	1.01
	DAP active (mmHg)	100 ± 1		102 ± 1	1.02
	PP inactive (mmHg)	28 ± 1	*	32 ± 1	1.14
	PP active (mmHg)	31 ± 1	*	34 ± 1	1.10
	HR inactive (beats min^-1^)	543 ± 4		544 ± 6	1.00
	HR active (beats min^-1^)	628 ± 4		634 ± 5	1.01

Telemetry data from 12 hour light and 12 hour dark periods were obtained from 33 PAR_2 _+/+ and 21 PAR_2 _-/- mice. Hemodynamic data were matched to locomotor activity status. Values are mean ± standard error of means. *P *values determined by multiple comparisons testing between strains during light and dark periods using 2 way ANOVA and Bonferroni post-hoc. *, *P *< 0.05; **, *P *< 0.01; ***, *P *< 0.001.

### Salt sensitivity of PAR_2 _-/- versus PAR_2 _+/+

**Figure 2:**
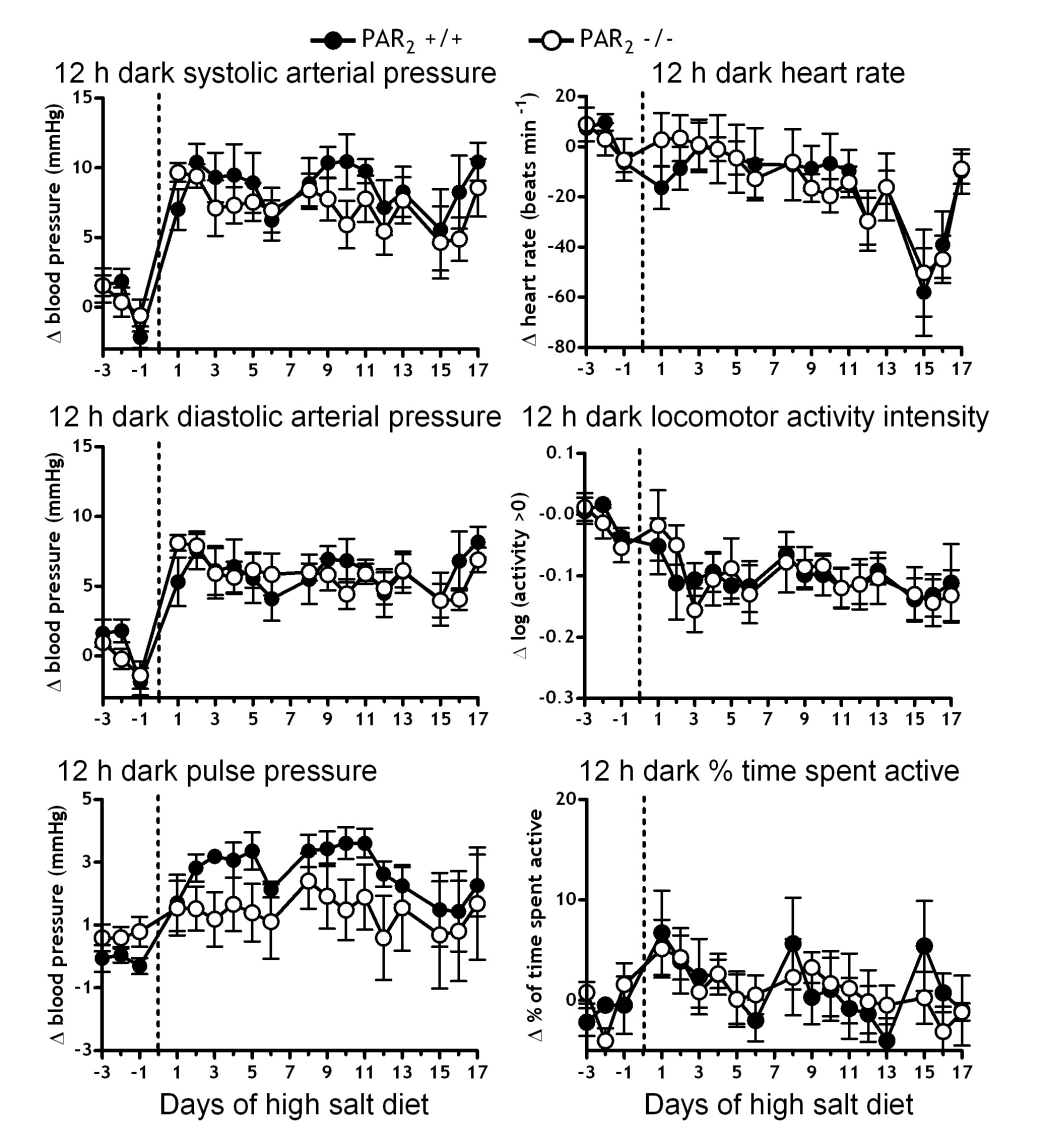
**Effect of high salt-diet feeding on PAR_2 _-/- versus PAR_2 _+/+ hemodynamics and locomotor activity during 12 h dark periods**. Variables were measured every 30 s for 18 days via radiotelemetry during a high salt diet (8% NaCl) ad libitium. Symbols represent the change in means of variables over the 12 h dark periods relative to baseline feeding with a normal salt diet (0.4% NaCl). n = 5 in each strain. 2 way repeated measures ANOVA indicated a significant difference (P < 0.05) for systolic and pulse pressures between PAR_2 _-/- and PAR_2 _+/+.

**Figure 3:**
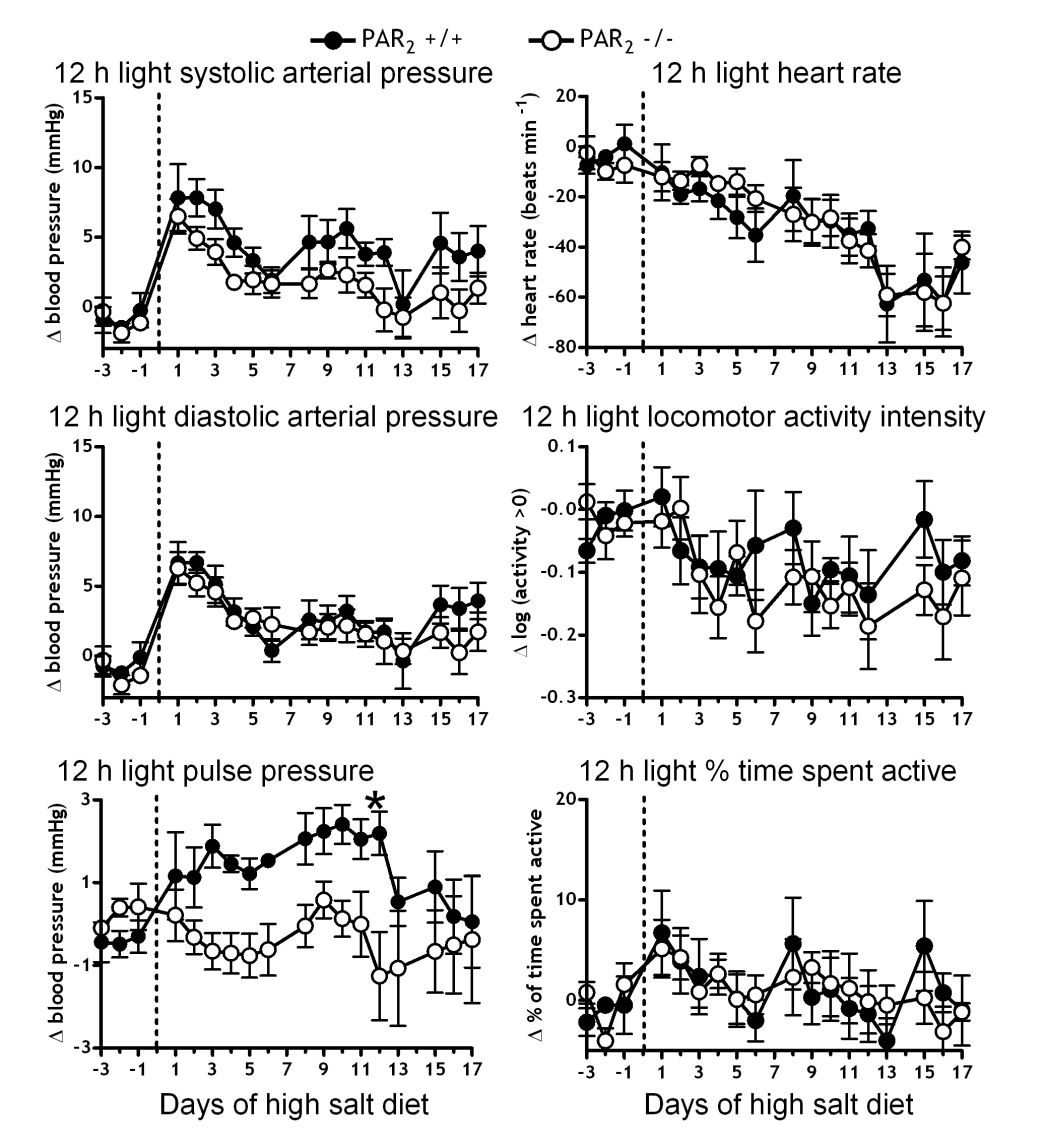
**Effect of high salt-diet feeding on PAR_2 _-/- versus PAR_2 _+/+ hemodynamics and locomotor activity during 12 h light periods**. Variables were measured every 30 s for 18 days via radiotelemetry during a high salt diet (8% NaCl) *ad libitium*. Symbols represent the change in means of variables over the 12 h light periods relative to baseline feeding with a normal salt diet (0.4% NaCl). n = 5 in each strain. 2 way repeated measures ANOVA indicated a significant difference (P < 0.05) for systolic and pulse pressures between PAR_2 _-/- and PAR_2 _+/+.* P < 0.05, PAR_2 _-/- Ang II compared to PAR_2 _+/+ Ang II, 2 way ANOVA and Bonferroni post-hoc test.

Both dark and light period BP were increased by ~3–10 mmHg by the high salt diet in both PAR_2 _-/- and PAR_2 _+/+ mice relative to the normal salt diet period (Figure 2 and Figure 3). 2 way ANOVA indicated a statistically significant effect of strain on elevation of SAP (*P *< 0.05) irrespective of night or day during the HS diet, but differences for specific time points were not resolved by Bonferroni post-hoc tests. However, in contrast to our initial hypothesis, the elevation of SAP by HS was less in PAR_2 _-/- than in PAR_2 _+/+. The elevation of DAP by HS diet was not different between the strains. 12 h dark period pressures remained elevated throughout the treatment period (Figure 2). In contrast, the 12 h light period pressures peaked on the first few days of high salt and then returned to baseline (Figure 3). Consistent with the finding of an effect of strain on systolic, but not on diastolic pressures, 2 way ANOVA indicated a significant effect of strain on the elevation of PP by the HS diet (P < 0.05) during both night and day, the salt-induced changes in PP being less in PAR_2 _-/- than in PAR_2 _+/+. We observed small 1–3 mmHg elevations of pulse pressures in PAR_2 _+/+ until day 13 of high salt diet. A significant (~3 mmHg) difference between strains was resolved by Bonferroni post-hoc for the 12 h light PP difference on day 12 (Figure 3). Both strains also exhibited similar decreased heart rates that plateaued at ~60 beats min^-1 ^below the RS diet period around day 13 of high salt diet.

### Angiotensin II effects on hemodynamics of PAR_2 _-/- and PAR_2 _+/+

**Figure 4:**
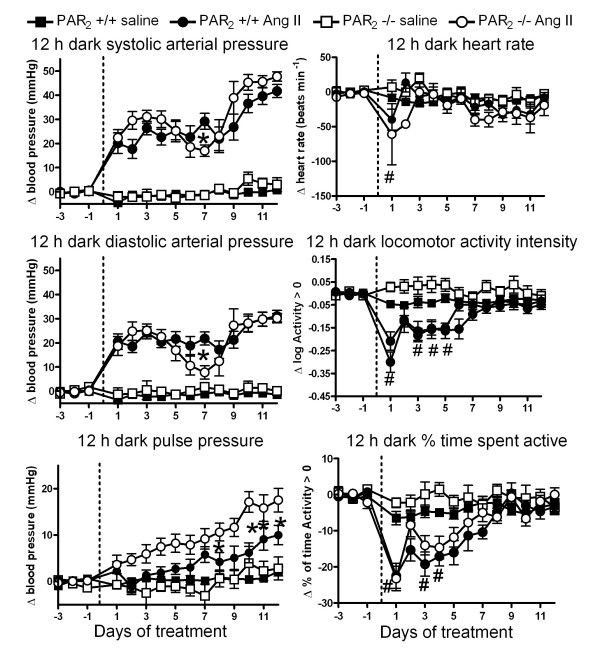
**Effect of angiotensin II and saline infusions on PAR_2 _-/- versus PAR_2 _+/+ hemodynamics and locomotor activity during 12 h dark periods**. Variables were measured every 30 s for 13 days via radiotelemetry during infusion s.c. with angiotensin II (1 μg/kg/min) or isotonic saline (0.5 μl/h). Symbols represent the change in means of variables averaged over 12 h dark periods relative to baseline periods prior to implant of micro-osmotic pumps. Data obtained from 13 PAR_2 _+/+ saline, 15 PAR_2 _+/+ Ang II, 7 PAR_2 _-/- saline and 9 PAR_2 _-/- Ang II. * P < 0.05, PAR_2 _-/- Ang II compared to PAR_2 _+/+ Ang II using 2 way ANOVA and Bonferroni post-hoc. # P < 0.05, Ang II-treated PAR_2 _+/+ and PAR_2 _-/- versus saline-treated groups, 2 way ANOVA and Bonferroni post-hoc test.

**Figure 5:**
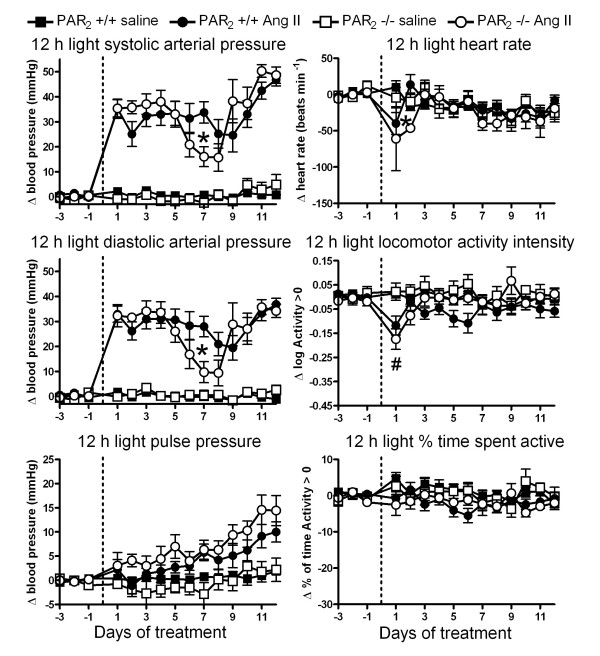
**Effects of angiotensin II and saline infusions on PAR_2 _-/- versus PAR_2 _+/+ hemodynamics and locomotor activity during 12 h light periods**. Variables were measured every 30 s for 13 days via radiotelemetry during infusion s.c. with angiotensin II (1 μg/kg/min) or isotonic saline (0.5 μl/h). Symbols represent the change in means of variables averaged over 12 h light periods relative to baseline periods prior to implant of micro-osmotic pumps. Data obtained from 13 PAR_2 _+/+ saline, 15 PAR_2 _+/+ Ang II, 7 PAR_2 _-/- saline and 9 PAR_2 _-/- Ang II. * P < 0.05, PAR_2 _-/- Ang II compared to PAR_2 _+/+ Ang II using 2 way ANOVA and Bonferroni post-hoc. # P < 0.05, Ang II-treated PAR_2 _+/+ and PAR_2 _-/- versus saline-treated groups, 2 way ANOVA and Bonferroni post-hoc test.

Angiotensin II infusion had relatively similar effects on BP in PAR_2 _-/- and PAR_2 _+/+, with systolic and diastolic pressures increasing by 20–55 mmHg over the 12 d infusion period (Figure 4 and 5). The interaction of time and treatment was significant for BP variables (2 way ANOVA *P *< 0.05). In both strains, the rise in BP tended to be biphasic, with an initial peak on days 1–4 being followed by a dip in BP before it again rose during the final days of the infusion. The intensity of the BP dip tended to be greater in PAR_2 _-/- mice, and this effect reached significance for all indices of BP recorded on the 7^th ^day of infusion. However, the initial and final BP levels produced by Ang II infusion did not differ between strains. Ang II infusion also led to a progressive increase in PP which tended to be greater in PAR_2 _-/- mice, an effect that reached statistical significance for data obtained during the 12 h dark period (Figure 4).

Ang II infusion was associated with similar transient reductions in HR, Act intensity and % time spent active in both strains. We did not observe any effect of micro-osmotic pumps containing saline on the variables of either strain of mouse relative to the baseline period (Figure 4 and Figure 5).

## Discussion

The main findings from our study were that gene knockout of PAR_2 _was associated with only a modest change in blood pressure phenotype. PAR-2 mice exhibited moderate elevation of systolic arterial and pulse pressures, yet no increase of diastolic arterial pressure, no increased responses to salt and only a subtle difference on the time course of the blood pressure responses to Ang II infusion.

We found small yet significant increases in blood pressures of PAR_2 _-/- mice which led us to conclude their phenotype is characterized by moderate elevation in systolic arterial and pulse pressures, but not diastolic arterial pressures. Our telemetry data comparisons of PAR_2 _-/- to the background genetic controls (C57BL/6J) provide the first description of blood pressures and heart rates in conscious PAR_2 _-/-. Previously it was reported that the acute hemodynamics (i.e. heart rates, blood pressures) of PAR_2 _-/- were not different than controls based on intracarotid catheter recordings under anaesthesia [4]. Using a radiotelemetry approach for blood pressure phenotyping in conscious unrestrained PAR_2 _-/- mice, we resolved a very small difference in hemodynamic variables. The magnitude of these differences were so small that the physiological impact may at first glance be considered inconsequential under baseline conditions and further dissection of its nature would appear to be unfeasible. Nevertheless the use of telemetry methodology allows us to conclude with a high degree of confidence that these subtle differences are in fact real effects of PAR_2 _-/-, being observed consistently in freely behaving conscious mice without the limitations that may be imposed by other methods (e.g. anesthetics, restraint, tail warming). Telemetry has been shown to be the most accurate of current techniques available for assessing BP in rodents and has received recommendation as the gold standard method for quantifying the BP phenotype in experimental animals [34]. We also made use of an advantage of radiotelemetrically acquired data to assess interactions between independent variables in order to explain the overall physiological phenotype. For example, analyses of the radiotelemetry data has the ability to dissect the indirect influence of genotype on hemodynamic variables that may arise as a consequence of behavioural changes occurring in the gene knockout. Locomotor activity changes resulting from an abnormal behaviour (hyperactivity) can result in changes to the distribution pattern of hemodynamic variables because conscious blood pressure in mice has been shown to be strongly influenced by locomotor activity [31,32]. No other method would likely have identified the small, but significant increase in HR of PAR_2 _-/- while active at night.

We found that HR in PAR_2 _-/- were relatively the same as PAR_2 _+/+ except during activity episodes at night. Differences in HR of PAR_2 _-/- and PAR_2 _+/+ during activity of the dark period coincided with similarly raised intensity levels of activity in the dark period (Table 2) which is usual for nocturnal animals. The significance of these findings is unknown, but could reflect a subtle effect of PAR_2 _-/- on autonomic nervous system reactivity or exercise intolerance.

Slightly higher PP in PAR_2 _-/- than in PAR_2 _+/+ could indicate reduced endothelial-derived vasodilator tone, reduced central arterial compliance resulting from morphological and composition changes of blood vessel walls (e.g. increased wall thickness, reduced internal diameters, collagen deposition) and/or increased cardiac stroke volumes in PAR_2 _-/-. Activation of PAR_2 _has numerous effects including endothelium-dependent vasodilation [32,35-38], so an elevated peripheral resistance in knockouts would be consistent with a subtle role played by PAR_2 _in modulating vascular tone. Endothelial NO synthase (eNOS) is considered a major modulator of peripheral resistance and the 24 h mean blood pressures of eNOS -/- mice were found to increase by ~15% relative to controls when measured by radiotelemetry [31]. The BP elevation observed for PAR_2 _-/- mice in the present study amounts to approximately one fourth of this level. Such differences in BP may be reduced by compensations from the time of conception so could explain the small size of effect of PAR_2 _-/- on baseline hemodynamics in mice even if PAR_2 _was a critical enzyme in blood pressure regulation. Theoretically, the small impact on BP could also underestimate the role played by PAR_2 _in modulating peripheral resistance (if cardiac output decreased in knockouts as part of compensation) or regulating local blood flow distribution.

For PAR_2 _to interact pathophysiologically with blood pressure control during high salt diet loading, there should be enzymes that mediate its activation that are linked to blood pressure regulation. The human kallikreins family of serine proteinases consists of several isoforms that have been found to activate PAR_2 _[39]. It was reported that renal kallikrein activity decreased in salt-sensitive hypertensive animals and renal excretion of kallikreins was increased by angiotensin II [20,21,40,41]. The majority of kallikreins biological actions are mediated through kinins, interaction with thrombin generation and the coagulation-cascade. However, the presence of residual non-kinin effects on blood pressure regulation by renal kallikreins during salt-loading has provided evidence of additional substrates [42]. In the current study we had hypothesized that PAR_2 _was one such substrate. As a possible contributor to the mechanism of residual blood pressure lowering activity by kallikreins during salt loading, we had expected that PAR_2 _-/- would have been more salt-sensitive relative to controls, but these mice were in fact less salt sensitive than controls. Even so, the impact of such small absolute differences in the salt-sensitivity of BP between PAR_2 _-/- and PAR_2 _+/+ would likely to be physiologically trivial, and suggests that PAR_2 _is unlikely to play a significant role in the regulation of salt balance. In both strains HR was lowered during HS diet, an observation that has been reported in humans [43]. The data do not exclude PAR_2 _interaction with non-blood pressure actions of HS diet that remain to be investigated.

In addition to HS diet, a condition where the renin-Ang II system would be downregulated, we also treated mice with Ang II infusion in order to investigate possible interaction with PAR_2 _on high Ang II acquired hypertension. At the end of 2 weeks, the effects of Ang II on BP were similar in PAR_2 _-/- and PAR_2 _+/+ mice. Collating the findings of classic physiology studies in multiple model systems, we expect that the immediate effects of elevating Ang II involve central nervous system and sympathetic nervous system activations and increases in peripheral resistance that are then followed later by mechanisms involving the resetting of kidney function and remodeling of the vasculature. Increasingly often there have been studies that have sought out links between inflammation and hypertension development involving angiotensin II. Studies to induce inflammatory responses in PAR_2 _-/- have resulted in mixed findings about pathological outcomes and our data support a main finding of a null effect on Ang II-acquired hypertension. Chronic high dose Ang II infusions over the period used in this study were shown to result in production of signs of vascular inflammation and endothelial dysfunction by the end-point, but have not been studied at the early time point (~7 day) at which we observed consistent differences in the trend for hemodynamic changes of PAR_2 _-/- [18,19]. We did not rule out the possibility that different results might be obtained in females. While a major interaction between Ang II and PAR_2 _appears inconsequential to BP in the long term, other pathological effects of high Ang II that arise independent of BP such as endothelial dysfunction or the effects of threshold BP changes by a lower dose of Ang II could be manifested differently in PAR_2 _-/-. Other characteristics of endothelium-dependent inflammatory responses which have been shown to be different have included the time to onset which was delayed in PAR_2 _-/- [9] and both leukocyte adhesion and neointima formation, which were reduced in PAR_2 _-/-[10].

In conclusion, PAR_2 _-/- mice exhibited a BP phenotype which was characterized by modest increases of systolic arterial and pulse pressures. The BP of PAR_2 _-/- is not more salt-sensitive than that of wild type mice, but differences in the time course of the BP responses to Ang II in PAR_2 _-/- and PAR_2 _+/+ may indicate the presence of a subtle influence of PAR_2 _on Ang II modulation of BP.

## Authors' contributions

JJM conceived and designed the study, supervised the planning and execution of experiments, acquisition of data, conducted data analysis and prepared the manuscript. SJH carried out the surgical and telemetry protocols and managed data collection. BVV provided guidance to study design, execution of technical protocols, data analyses and preparation of the manuscript. All authors have read and approved the final draft of the manuscript. Granting agencies did not contribute to study design, data collection, analyses, interpretation, manuscript writing or decision to submit the manuscript for publication.
